# Altered movement during single leg hop test after ACL reconstruction: implications to incorporate 2-D video movement analysis for hop tests

**DOI:** 10.1007/s00167-018-4893-7

**Published:** 2018-03-16

**Authors:** Wouter Welling, Anne Benjaminse, Romain Seil, Koen Lemmink, Alli Gokeler

**Affiliations:** 1 Medisch Centrum Zuid, Sportlaan 2-1, 9728 PH Groningen, The Netherlands; 2 Center for Human Movement Science, University Medical Center Groningen, University of Groningen, Antonius Deusinglaan 1, 9713 AV Groningen, The Netherlands; 3 School of Sport Studies, Hanze University Groningen, Zernikeplein 17, 9747 AS Groningen, The Netherlands; 4 Département de l’Appareil Locomoteur, Centre Hospitalier de Luxemburg, 4 Rue Nicolas Ernest Barblé, 1210 Luxembourg, Luxembourg; 5 Sports Medicine Research Laboratory, Luxembourg Institute of Health, 4 Rue Nicolas Ernest Barblé, 1210 Luxembourg, Luxembourg

**Keywords:** Anterior cruciate ligament, Hop tests, Jump landing, Return to sport, Movement analysis

## Abstract

**Purpose:**

There is a lack of objective factors which can be used in guiding the return to sport (RTS) decision after an anterior cruciate ligament reconstruction (ACLR). The purpose of the current study was to conduct qualitative analysis of the single leg hop (SLH) in patients after ACLR with a simple and clinical friendly method and to compare the possible difference in movement pattern between male and female patients.

**Methods:**

Sixty-five patients performed the single leg hop (SLH) test at 6.8 ± 1.0 months following isolated ACLR. Digital video camcorders recorded frontal and sagittal plane views of the patient performing the SLH. Knee flexion at initial contact (IC), peak knee flexion, knee flexion range of motion (RoM), and knee valgus RoM were calculated. In addition, limb symmetry index (LSI) scores were calculated.

**Results:**

No differences were found in movement pattern between males and females. Movement analysis revealed that males had a decrease in knee flexion at IC (*p* = 0.018), peak knee flexion (*p* = 0.002), and knee flexion RoM (*p* = 0.017) in the injured leg compared to the non-injured leg. Females demonstrated a decrease in peak knee flexion (*p* = 0.011) and knee flexion RoM (*p* = 0.023) in the injured leg compared to the non-injured leg. Average LSI scores were 92.4% for males and 94.5% for females.

**Conclusions:**

Although LSI scores were > 90%, clinical relevant altered movement patterns were detected in the injured leg compared to the non-injured leg. Caution is warranted to solely rely on LSI scores to determine RTS readiness.

**Clinical trial registry name and registration:**

The University of Groningen, ID 2012.362.

**Level of evidence:**

III.

## Introduction

Although an anterior cruciate ligament reconstruction (ACLR) is considered as a successful procedure [2], the rate of return to sport (RTS) in patients after ACLR is relatively low [14]. Over two-third of patients do not return to their pre-injury level of sport 1 year after surgery [2]. Unfortunately, RTS are associated with ACL re-injury rates reported between 6–20% [6, 48, 49, 57]. The majority of ACL re-injuries (74%) occur within the first 2 years after RTS [24].

The reason of this high rate of re-injuries after ACLR is multifactorial [12]. One of the contributing factors may be related movement asymmetries after ACLR, which have been directly implicated in the risk for ACL-re-injury [8]. These deficits may have been present prior to injury and exacerbated by the surgical procedure. A critical moment towards the end of an extensive course of rehabilitation is the clearance by physicians and rehabilitation specialists to release athletes after ACLR to full RTS [2, 56].

To determine the RTS readiness, the most commonly assessments are clinical, strength, performance-based functional (like hop testing) and self-reported knee function [30]. It is common to calculate a limb symmetry index (LSI) defined as the hop test performance of the injured leg divided by the hop test performance of the non-injured leg × 100% [1]. LSI > 90% are often used as cut-off scores for RTS [18, 26]. Research shows that RTS decisions are frequently based on subjective criteria [4] and quantitative analysis of functional tests (distance, time or LSI) [32, 39], while outcomes related to the quality of movement are not captured [50]. The current method may not be sensitive enough to detect deficits related to ACL re-injury risk [18].

Research suggests that decreased knee flexion angles will potentially increase the risk of a re-injury, since a more stiff landing will generated more forces on the ACL [28]. Decreased knee flexion compared to the non-injured leg has been reported for hop tests 7 months after ACLR [55]. These findings may explain the relatively high re-injury rates that are found in ACLR patients [5] and show that the quality of the movement is essential in ACL rehabilitation [38, 48]. Males and females differ in neuromuscular movement patterns and it is suggested that females have a two to three times higher ACL injury risk compared to males [13]. More specific, females show more knee valgus range of motion (RoM) during landing which can potentially increase knee abduction moment [20, 27].

Two decades ago, the need to include movement quality rather than quantitative parameters was already proposed [40]. Motion analysis methods are often 3D motion capture systems which are time-consuming methods to detect movement asymmetries [16, 31, 55]. There is need for simple, clinical friendly methods to analyze movement quality to detect possible asymmetrical movement patterns after ACLR to aid in decision-making of the athlete to RTS [35, 52, 53]. Therefore, the purpose of the current study was to conduct qualitative analysis of the single leg hop (SLH) in patients after ACLR with a simple and clinical friendly method, and to compare the possible difference in movement pattern between male and female patients. It was hypothesized that altered knee movement patterns are found in the injured leg compared to the non-injured leg for both males and females, and that patients that passed the LSI > 90% criteria will demonstrate altered knee movement patterns in the injured leg compared to the non-injured leg. In addition, it was hypothesized that males and females differ in movement patterns around 6 months after isolated ACLR.

## Materials and methods

Sixty-five patients (45 males, 20 females) participated in the current study. All patients rehabilitated in the same physical therapy in Groningen, The Netherlands. Descriptive data can be found in Table 1. Inclusion criteria for the patients were: (1) between 6 and 8 months after ACLR, (2) age between 16 and 45 years, (3) primary isolated ACL lesion and no major meniscal or cartilage lesion. An arthroscopic ACLR with anteromedial portal technique was performed on all patients by the same two orthopedic surgeons. All patients underwent a standardized rehabilitation protocol [18] but did not finished the protocol at time of data collection yet. In the first 6 weeks after surgery, the focus in rehabilitation was to reduce inflammation and swelling and to restore full knee extension, gait training, and neuromuscular training addressing full body. Neuromuscular training continued after 6 weeks, with more advanced drills; and muscle strengthening and endurance training were added. Muscle hypertrophy strengthening was started after 12 weeks, and running activities and jumping tasks were added. After 24–36 weeks, the focus was more on plyometric activities and running/cutting drills. In addition, sport-specific agility drills on the field were added [18].

**Table 1:** Demographics of the ACLR patients

	*n*	Age (years)	Mass (kg)	Time post-surgery (months)	Graft type	IKDC	Quadriceps strength (Nm) (LSI)	Hamstring strength (Nm) (LSI)
Males	45	25.4 ± 7.2	79.5 ± 10.3	6.7 ± 1.0	HT(30), PT(13), DT(2)	81.4 ± 8.3 (52–97)	Injured leg: 221.4 ± 54.1, Non-injured leg: 254.6 ± 55.0 (87.2 ± 11.7%)	Injured leg: 136.4 ± 30.5, Non-injured leg: 144.1 ± 29.9 (95.0 ± 11.8%)
Females	20	22.8 ± 6.5	67.7 ± 9.9	6.1 ± 1.1	HT(17), PT(3)	80.4 ± 8.9 (52–93)	Injured leg: 157.4 ± 37.7, Non-injured leg: 177.6 ± 35.0 (88.6 ± 11.0%)	Injured leg: 95.2 ± 21.1, Non-injured leg: 101.9 ± 18.9 (93.1 ± 7.4%)

Mean ± SD

*HT* hamstring tendon, *PT* patellar tendon, *DT* tendon allograft, *IKDC* International Knee Documentation Committee Subjective Knee Form

### Procedures

**Fig. 1:**
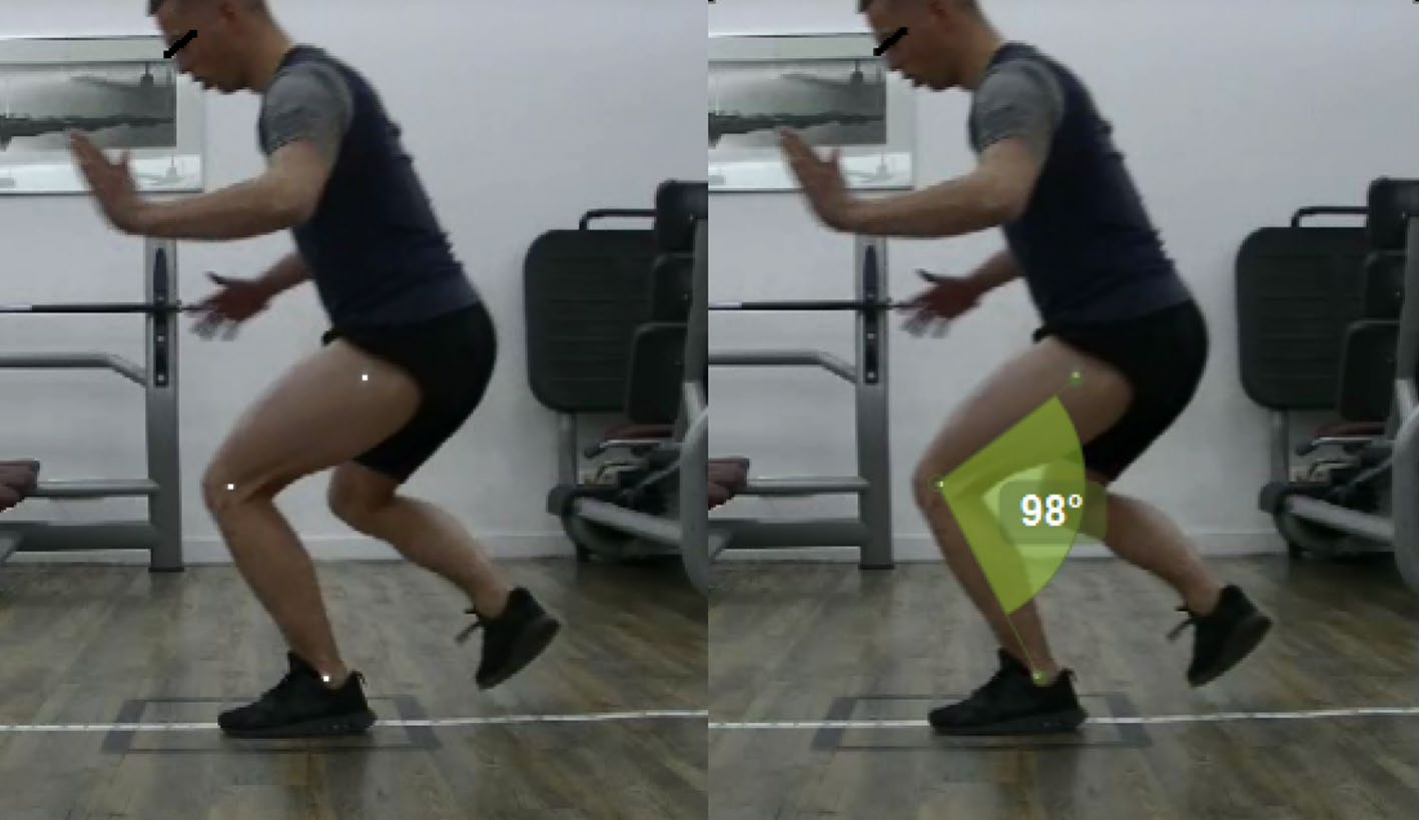
Example of a patient performing the SLH from the sagittal plane, with markers on the trochanter major, lateral epicondyle of the femur and the lateral malleoli (left), and the knee flexion angle calculation (right)

The functional test used in the current study was the single leg hop (SLH) test, which has been shown high reliability (ICC = 0.97) [23]. The SLH was performed as previously reported [15, 19, 38]. Before testing, patients completed a 5 min warm-up on a stationary exercise bike. Patients were given a general instruction about the jumping task “Stand on one leg, jump as far possible and land on the same leg.” All patients practiced three times with each leg. The SLH was deemed correctly if the patient was able to achieve maximal hop distance while maintaining balance for at least 2 s after landing. Patients started jumping with their non-injured leg; and for each leg, three successful jumps were recorded. The SLH was recorded with two commercially available video cameras (60 Hz, JVC Everio GZ-E105BE) as previously reported [15]. The set-up is very similar to a previous published report that demonstrated that this is a valid and reliable (ICC = 0.91) [33] method to identify potentially high-risk movement patterns during a jump-landing task. Before testing, markers were placed on the trochanter major, lateral epicondyle of the femur, the lateral malleoli (Fig. 1) and the center of the patella. Patients wore their own sport shoes during the tests. Patients were excluded if (1) they had pain during the test, (2) presence of swelling of the injured knee, or (3) feeling of instability in the injured knee. Data collection took place between April 2015 and December 2016 in an outpatient physical therapy clinic.

An isokinetic device (Biodex System 3; Biodex Medical Systems, Inc., Shirley, NY) was used to test quadriceps and hamstring strength for both legs at a velocity of 60°/s, with five maximal concentric repetitions for flexion and extension. The non-injured leg was tested first. After the strength testing, the patients were asked to fill in the International Knee Documentation Committee Subjective Knee Form (IKDC) which is a knee-specific outcome measure, containing 18 questions, pertinent to a variety of knee conditions for assessing symptoms, function, and sports activity [21].

### Data reduction

LSI values were calculated by dividing the jumping distance with the injured leg by the jumping distance with the non-injured leg × 100%. Leg dominance was not considered, since research suggested that there are no relevant difference in movement pattern between the dominant and the non-dominant leg; and therefore, the non-injured leg can be used as a reference for the injured leg [46]. Movement analysis was obtained from video data and analyzed by the same researcher who has 5 years of experience in analyzing video data. The researcher was blinded for the injured leg as videos of the SLH were analyzed in a random order. Video analyzing software (Kinovea 0.8.15, ICC = 0.920–0.995) [12] was used and videos were played frame-by-frame to collect the primary outcome variables: knee flexion at initial contact (IC), peak knee flexion, knee flexion RoM, and knee valgus RoM. The knee flexion angle was defined as the angle between the the trochanter major, the lateral epicondyle of the femur, and the lateral malleoli of the stance leg. Knee flexion at IC was defined as the knee flexion angle at the instant the foot contacted the floor. Peak knee flexion was defined as the maximal knee flexion angle during landing. Knee flexion RoM was calculated as the difference between knee flexion at IC and peak knee flexion. In the frontal plane, knee valgus RoM was calculated as the movement of the center of the patella between knee valgus at IC and peak knee valgus. The average of three jumps was used for the analysis. IKDC scores were compared to *n* age and gender normative IKDC data [18]. The study protocol was approved by the Medical Ethical Committee (ID 2012.362) of the University of Groningen, and informed consent was obtained from all patients prior to data collection.

### Statistical analysis

A power analysis (*G*Power, Version 3.1.7) was used to calculate the required sample size. With an effect size of 0.25 (medium effect ANOVA) and an alpha of 0.05, 34 patients were required to obtain a power of 0.80 based on the primary outcome variables knee flexion at IC, peak knee flexion, knee flexion RoM, and knee valgus RoM [7]. In total, 65 patients were included. All data were normally distributed as analyzed with SPSS version 20 (SPSS 244 Inc, Chicago, IL). To determine differences between sex (female and male) and legs (non-injured leg and the injured leg), a 2 × 2 ANOVA was conducted. A clinical meaningful difference was defined as 3° for knee flexion angle and 4.15 cm for knee valgus RoM [30, 41]. An additional 2 × 2 ANOVA was conducted to investigate if there was a difference in knee flexion at IC, peak knee flexion, knee flexion RoM, and knee valgus RoM in patients who passed the LSI criteria and patients who did not pass the LSI criteria. Statistical significance was set at *p* < 0.05 level of confidence. A Pearson correlation analysis was conducted to determine the correlation between the outcome variables. Effect sizes (ES) were calculated and Cohen’s d values are reported as a measure of ES, where 0.20 ≤ *d* ≤ 0.50, 0.50 ≤ *d* ≤ 0.80, and *d* ≥ 0.80 represent small, moderate, and large effects. IKDC values of the patients were compared with normative IKDC values from the previous research [18].

## Results

**Fig. 2:**
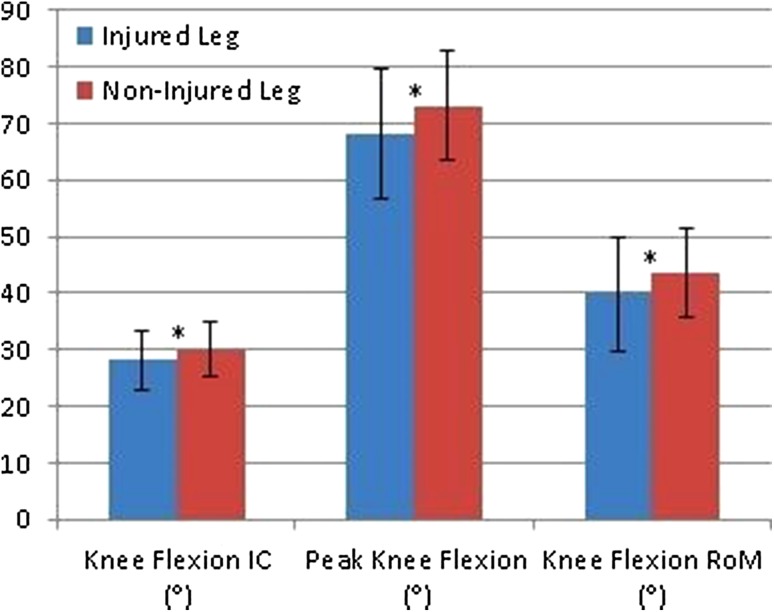
Graphical representation of mean knee flexion angles in male patients including standard deviations (*significant difference). *IC* initial contact; ° degrees; *RoM* range of motion

**Fig. 3:**
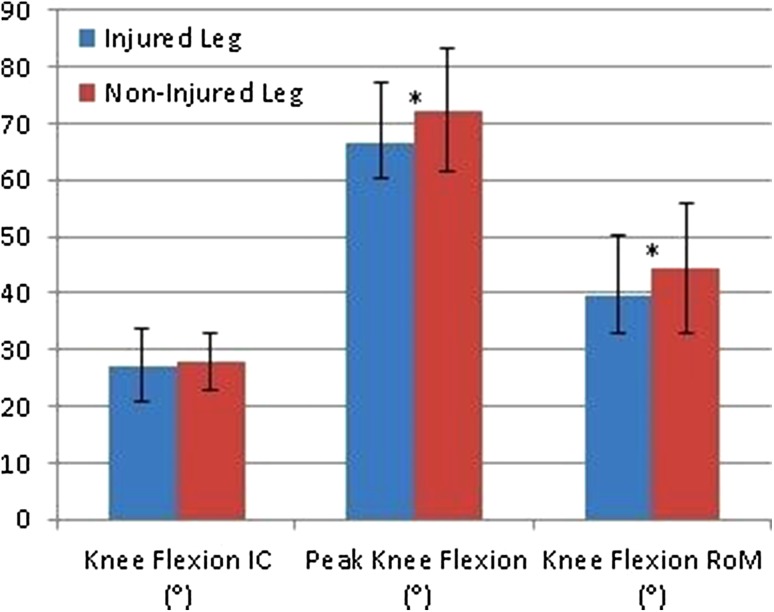
Graphical representation of mean knee flexion angles in females patients including standard deviations (*significant difference). *IC* initial contact; ° degrees; *RoM* range of motion

Results are presented in Table 2 and Figs. 2 and 3. Mean LSI scores were 92.4 ± 8.5% for males and 94.5 ± 6.4% for females, respectively. In total, 47 patients (72.3%, 31 males and 16 females) passed the LSI > 90% criteria. Between sex analysis showed that males jumped significantly further with both their non-injured leg and injured leg compared to females (injured leg: *p* = 0.001, non-injured leg: *p* < 0.001). No significant differences between males and females were found in movement pattern.

**Table 2:** Results of the analyses within males and females

	Injured leg mean ± SD	Non-injured leg mean ± SD	95% CI	*p* value
Males				
Jumping distance (cm)	158.2 ± 25.8	171.0 ± 22.4	− 16.9 to − 8.7	< 0.001*
Knee flexion IC (°)	28.2 ± 5.3	30.1 ± 4.8	− 3.5 to − 0.3	0.018*
Peak knee flexion (°)	68.1 ± 11.5	73.2 ± 9.6	− 8.4 to − 1.9	0.002*
Knee flexion RoM (°)	40.0 ± 10.0	43.6 ± 8.9	− 6.6 to − 0.7	0.017*
Knee valgus RoM (cm)	1.5 ± 1.5	1.1 ± 1.2	− 0.9 to 0.0	n.s.
Females				
Jumping distance (cm)	131.0 ± 19.7	138.6 ± 17.9	− 11.6 to − 3.5	0.001*
Knee flexion IC (°)	27.2 ± 6.7	27.9 ± 5.1	− 3.3 to 1.9	n.s.
Peak knee flexion (°)	66.7 ± 10.4	72.3 ± 10.8	− 9.7 to − 1.4	0.011*
Knee flexion RoM (°)	39.6 ± 10.8	44.4 ± 11.3	− 9.0 to − 0.7	0.023*
Knee valgus RoM (cm)	1.4 ± 1.4	1.6 ± 0.6	− 1.2 to 0.6	n.s.

*cm* centimeter, *IC* initial contact, ° degrees, *RoM* range of motion, *CI* confidence interval, *n.s*. not significant

*Significant difference

Patients who passed the LSI > 90% criteria (*n* = 47) had significant decreased peak knee flexion in their injured leg compared to their non-injured leg (*p* = 0.027). Patients who did not pass the LSI > 90% criteria (*n* = 18) had a significant decrease in knee flexion at initial contact (*p* = 0.007), decreased peak knee flexion (*p* < 0.001), and decreased knee flexion RoM in the injured leg compared to the non-injured leg (*p* = 0.002). Patients who passed the LSI > 90% criteria (*n* = 47) showed more knee flexion at the initial contact with their injured leg (*p* = 0.004) and more peak knee flexion with their injured leg (*p* = 0.005) compared to patients who did not pass the LSI > 90% (*n* = 18). Furthermore, 27 of all patients (41.5%) had a clinically relevant difference in knee flexion at the initial contact, 39 of all patients (60.0%) had a clinically relevant difference in peak knee flexion, and 38 of all patients (58.5%) had a clinically relevant difference in knee flexion ROM in their injured leg compared to the non-injured leg.

For males, small significant correlations were found for the injured leg between knee flexion at the initial contact and jumping distance (0.30, *p* = 0.047), between peak knee flexion and quadriceps strength (0.33, *p* = 0.025), between peak knee flexion and hamstring strength (0.34, *p* = 0.021), and between jumping distance and quadriceps strength (0.42, *p* = 0.004). For females, moderate significant correlations were found between jumping distance and hamstring strength for the non-injured leg (0.58, *p* = 0.008) and for the jumping distance and quadriceps strength in the injured leg (0.56, *p* = 0.011). In addition, females showed small significant correlations between the peak knee flexion and quadriceps strength (0.47, *p* = 0.037) and between jumping distance and hamstring strength (0.49, *p* = 0.029) in the injured leg.

The mean IKDC score of the patients was 81.08 ± 8.45 (males 81.41 ± 8.33; females 80.35 ± 8.90). No difference was found in IKDC score between males and females.

## Discussion

The main finding of the current study is that patients demonstrated altered movement patterns after ACLR. No differences were found in movement pattern between males and females. However, males showed decreased knee flexion at IC, decreased knee flexion RoM, and decreased peak knee flexion in the injured leg compared to their non-injured leg. Females demonstrated a decrease in peak knee flexion and a decrease in knee flexion RoM in their injured leg compared to their non-injured leg. Patients that passed the LSI > 90% criteria showed decreased peak knee flexion in the injured leg compared to the non-injured leg. However, patients that passed the LSI > 90% criteria showed more symmetrical movement compared to patients that not passed the LSI > 90%. Only small correlations were found between jumping distance and movement technique, indicating that both the quantitative data (jumping distance) and qualitative data (movement technique) are not strongly related and should be analyzed. These findings suggest that movement technique should be analyzed in RTS decisions, since altered biomechanics after ACLR could be a risk factor for ACL re-injury [36, 43]. The method used in the current study is relatively simple and clinical friendly for analyzing movement kinematics in patients after ACLR.

A soft landing with increased knee flexion is more conducive to prevent ACL injury than a stiff landing [28]. Increased knee flexion angles will generate more center of mass (CoM) displacement and, therefore, increase the potential to absorb the ground reaction forces (GRF) [53]. Furthermore, increased knee flexion angles will generate less forces on the ACL in the sagittal plane and, therefore, potentially decrease the risk of a re-injury [28]. Patients demonstrated less knee flexion angles in the injured leg compared to the non-injured leg, which are clinically relevant, since the differences are greater compared to the minimal clinical difference (3°) [34, 41]. These findings are in line with the previous research investigating differences in knee flexion angles after ACLR [17, 31, 44, 50, 55]. More in detail, Orishimo et al. [31] found a decreased knee flexion RoM in the injured leg compared to the non-injured leg (35.7° versus 43.4°) around 7 months after ACLR. These findings are similar to our knee flexion RoM results for the injured leg and non-injured leg (males 39.9° versus 43.6°; females 39.6° versus 44.4°). Furthermore, the results of the current study are in line with other studies which found altered movement patterns in after ACLR in functional movements like for example a single leg squat jump [9] and a drop jump landing [11]. Less optimal movement quality during functional movements can increase the re-injury risk [10, 47].

Knee valgus is a risk factor for an ACL re-injury as high valgus loading can increase relative ACL strain and may be of levels high enough to bring the ACL about to failure [3, 36, 51]. In addition, knee valgus in combination with decreased knee flexion RoM results in even more forces on the ACL in the injured leg, resulting in a higher risk of re-injury [36]. In the current study, no significant differences were found in knee valgus RoM in the injured leg compared to their non-injured leg for both males and females. In addition, no difference was found in knee valgus RoM between males and females. This is a surprising result, since the study of Malinzak et al. [27] found more knee valgus in females compared to males during athletic tasks. The limited knee valgus RoM in the current study can possibly be explained by the fact that all patients were trained to land with limited knee valgus RoM during their rehabilitation. In addition, they focused on maintaining balance for 2 s after the SLH and, subsequently, probably did not perform the SLH with maximal effort and, therefore, show limited knee valgus RoM. These findings are in line with the previous research which found no differences in knee valgus RoM between an injured leg and a non-injured leg in a single leg hop test after an ACLR [13].

In clinical care, an LSI of > 90% of hop tests is used as pass criteria for RTS after ACLR [18, 37]. The mean LSI’s scores in the current study were 92.4 ± 8.5% for males and 94.5 ± 6.4% for females. In total 31 males (68.9%) and 16 females (80%) passed the LSI > 90% criteria. Although patients passed the LSI > 90% criteria, they had a significant decreased peak knee flexion in their injured leg compared to their non-injured leg. Relying solely on the use of LSI > 90% for athletes who return to pivoting/contact type sports may, therefore, be questioned [45]. Our study results showed significant differences in peak knee flexion angles, which indicates that suboptimal landings strategies are still present around 6 months after ACLR. A possible reason for the difference in movement pattern could be the difference in quadriceps strength (LSI 87%) [25, 42, 46]. However, only small correlations were found between quadriceps strength and movement pattern in the current study, indicating that quadriceps strength and movement pattern are not strongly related. There is a lack of objective factors which can be used in guiding the RTS decision [2, 4] and the method used in the current study is a simple, clinical friendly method to detect possible altered movement patterns which can aid in RTS decisions.

The IKDC has been regarded as a measure of successful outcome after ACLR [21, 22]. Patients in the current study had an average IKDC score of 81.1 ± 8.5 (males 81.4 ± 8.3; females 80.4 ± 8.9). Research shows that an IKDC score within 15th percentile of an age-matched, uninjured group is a reliable cut-off score for representing normal variance [18]. The majority of our patients scored below these cut-off scores (males; 89.7–85.1, females; 83.9–82.8; [18]), which indicates less subjective function after ACLR compared to healthy controls.

There are some limitations that should be acknowledged. In the current study, 2D cameras were used to analyze lower extremity movement technique. The studies mentioned above [16, 31, 54, 55] used 3D motion capture systems, which are more time-consuming but more accurate methods compared to the relatively simple, clinical friendly method used in the current study. However, the use of 2D cameras is a practical, and a relatively simple way of analyzing movement kinematics and the use of 2D cameras have shown to be valid and reliable in analyzing lower extremity kinematics with a standard error of measurement (SEM) between 2.7° and 3.2° maximally [29]. In addition, the current study was focused on testing in an isolated and clinical environment instead of an open environment which is more representable for the eventual knee function that needs to be achieved [10]. Another limitation in the current study was the fact that there was some difference in time after surgery (range between 5.9 and 7.6 months) in the study population. In addition, the relatively wide age range (16–45) of our patients could have impact on the study results. Finally, detailed surgery data (graft fixation, graft components, and bundle configuration) were not taken into account in the current study.

## Conclusion

Of the patients who passed LSI scores > 90% for the SLH, altered movement patterns were present in 60%. On the basis of these results, clinicians should consider assessment of limb quality of movement by video taping the single leg hop test and analyze movement kinematics with a relatively simple and clinical friendly method to aid in the decision-making process for RTS. Persistent abnormal movement patterns increase the risk for an ACL re-injury.
